# 898. Effectiveness and Tolerability of DTG + 3TC in Clinical Practice: Evidence in PLHIV from Real-world Data

**DOI:** 10.1093/ofid/ofab466.1093

**Published:** 2021-12-04

**Authors:** Lee A Evitt, Rahul Kumar, Rahul Kamath, Diwakar Jha, Daniel Parks, Jean A van Wyk, Annemiek de Ruiter

**Affiliations:** 1 VIIV Healthcare, London, UK; 2 GlaxoSmithKline Knowledge Centre, Gurgaon, Haryana, India; 3 GlaxoSmithKline Knowledge Centre, Gurugram, Haryana, India; 4 GlaxoSmithKline Knowledge Center, Gurgaon, Haryana, India; 5 GlaxoSmithKline, Collegeville, PA; 6 ViiV Healthcare, London, UK

## Abstract

**Background:**

Randomized controlled trials have shown dolutegravir (DTG) + lamivudine (3TC) to be an efficacious, well-tolerated and durable regimen for treatment-naive and treatment-experienced people living with HIV (PLHIV). Several observational studies have also concluded that it is effective in clinical practice. The objective of this meta-analysis was to estimate effectiveness and tolerability of DTG + 3TC in PLHIV by combining real-world evidence from clinical practice.

**Methods:**

A systematic literature review using PubMed and Embase plus 24 regional and international conferences was conducted between January 2013 and December 2020 to identify studies of DTG + 3TC in treatment-experienced and treatment-naive PLHIV in clinical practice. Eligible published articles reporting virologic suppression, virologic failure and discontinuations at Weeks 48 and 96 were identified and extracted. Identified studies were included if they had an acceptable level of publication bias and heterogeneity determined using funnel plots and I^2^ statistics, respectively. One-arm meta-analyses using the DerSimonian and Laird method were conducted to estimate effect sizes for outcomes of interest for DTG + 3TC.

**Results:**

One study of DTG + 3TC was identified reporting outcomes of interest at time points of interest in treatment-naive PLHIV, hence no meta-analysis was undertaken in this population. Eight studies (N=2366 PLHIV) undertaken in Europe reported data on treatment-experienced, virologically suppressed PLHIV on outcomes of interest at time points of interest (not all endpoints/time points were reported by all studies). The meta-analysis of available data from these 8 studies showed that among PLHIV switching to DTG + 3TC treatment, ≥ 95% maintained virologic suppression (per protocol) with ~1% virologic failures on DTG + 3TC at Weeks 48 and 96. Five of the 8 studies reported resistance data. Among participants with baseline resistance testing, no treatment-emergent integrase strand transfer inhibitor resistance mutations were observed.

Table. Meta-analysis Results in Treatment-Experienced PLHIV: Proportion with Virologic Failure, Virologic Suppression, and Discontinuations at Weeks 48 and 96

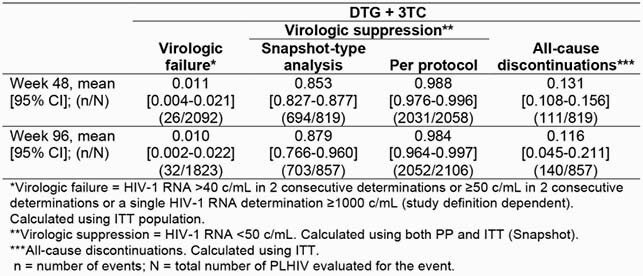

**Conclusion:**

DTG + 3TC is an effective, tolerable and durable antiretroviral regimen with low rates of discontinuation in treatment-experienced PLHIV in clinical practice.

**Disclosures:**

**Lee A. Evitt, MSc Health Economics**, **ViiV Healthcare** (Employee, Shareholder of GSK) **Rahul Kumar, M Pharmacy**, **GlaxoSmithKline** (Employee) **Rahul Kamath, PharmD**, **GlaxoSmithKline** (Employee) **Diwakar Jha, Masters in Pharmaceutical Sciences**, **GlaxoSmithKline** (Employee) **Daniel Parks, PhD**, **GlaxoSmithKline** (Employee, Shareholder) **Jean A. van Wyk, MB,ChB**, **GlaxoSmithKline** (Shareholder)**ViiV Healthcare** (Employee) **Annemiek de Ruiter, MBBS FRCP**, **GlaxoSmithKline** (Shareholder)**ViiV Healthcare** (Employee)

